# A novel insertional mutation in the connexin 46 (*gap junction alpha 3*) gene associated with autosomal dominant congenital cataract in a Chinese family

**Published:** 2013-04-05

**Authors:** Dingan Zhou, Hongyun Ji, Zhiyun Wei, Luo Guo, Yanpeng Li, Teng Wang, Yu Zhu, Xingran Dong, Yang Wang, Lin He, Qinghe Xing, Lirong Zhang

**Affiliations:** 1 Children’s Hospital and Institutes of Biomedical Sciences, Fudan University, Shanghai, P.R. China; 2 Yongchuan Hospital, Chongqing Medical University, Chongqing, P.R. China; 3 Shanghai Aier Eye Hospital, Shanghai, P.R. China; 4 Bio-X Institutes, Key Laboratory for the Genetics of Developmental and Neuropsychiatric Disorders (Ministry of Education), Shanghai Jiao Tong University, Shanghai, P.R. China; 5 The First Affiliated Hospital, Zhengzhou University, Zhengzhou, P.R. China; 6 Institutes of Biomedical Sciences, the Tian Lab of the Institute of Biostatistics, Fudan University, Shanghai, P.R. China; 7 Department of Pharmacology, School of Medicine, Zhengzhou University, Zhengzhou, P.R. China

## Abstract

**Purpose:**

To identify the genetic defect associated with autosomal dominant congenital cataract (ADCC) in a Chinese family, in which 11 individuals across four generations are affected with coralliform cataract.

**Methods:**

Exome sequencing was performed in two of the ADCC-affected family members to scan for potential genetic defects. Sanger sequencing was used to verify these defects in the whole family.

**Results:**

By combining whole exome sequencing and Sanger sequencing, the genetic defect was revealed to be a insertion of a cytosine after coding nucleotide 1,361 (1361insC) in the gap junction alpha 3 (*GJA3*) gene, causing a frameshift at codon 397 (p.Ala397Glyfs×71). This frameshift mutation cosegregates with the ADCC-affected pedigree members, but is absent in unaffected relatives and 100 normal individuals.

**Conclusions:**

A 1361 insC mutation in the C-terminus of *GJA3* is found to be associated with autosomal dominant congenital coralliform cataract. This finding is similar to that of a previous publication, thus providing further evidence that the *GJA3* C-terminal domain is also its mutation area, and further expanding the mutation spectrum of *GJA3* in association with congenital cataract.

## Introduction

Congenital cataract is a clinically heterogeneous disorder of opacity of the crystalline lens, usually presenting at birth (congenital), or during infancy, childhood, or adolescence. Congenital and infantile forms of cataract result in the blurring of images on the immature retina, which is a clinically important cause of impaired form vision development [1]. The prevalence of congenital cataract is estimated to be 0.01%–0.06%, acting as the second major cause of blinding eye disease in children [2,3]. Congenital cataract exhibits high clinical and genetic heterogeneity. One-third of congenital cataract cases are considered to be hereditary, and most occur in a nonsyndromic autosomal dominant fashion [4].

Connexins are components of gap junctions, which are membrane specializations containing intercellular channels that allow for the passage of ions and low–molecular weight molecules between adjacent cells. Disruption of either of the two connexin genes, *connexin 46* (*CX46*, *gap junction alpha 3* [*GJA3*]) and *connexin 50* (*CX50*, *GJA8*), which are the major components of mammalian lens fiber cells, results in cataracts in mice. Mutations in connexin genes are often associated with pulverulent (dust-like) nuclear opacities; *GJA3* and *GJA8* mutations account for approximately 20% of nonsyndromic familial cataract cases [5]. *GJA3* encodes a 435 amino acid protein that has been identified as one of the disease-causing genes of autosomal dominant congenital cataract (ADCC), and at least 15 mutations of *GJA3* have been reported to cause ADCC [6-17]. Most of these GJA3 protein mutations have been mapped in the NH_2_-terminus, transmembrane domain, extracellular domain, and extracellular loop. Less information is available regarding the insertional mutation of *GJA3*, with the exception of the CX46S380fs mutation [8].

In the reported mutations of *GJA3* involved in congenital cataract, only the functions of the CX46S380fs and CX46N63S mutations have been identified in the pathogenesis of the disease. In this study, we localized the mutation site of a Chinese family with congenital coralliform cataract to locus 13q11 around the known ADCC locus, and found a novel 1361 insC (CX46A397fs) mutation in *GJA3* exhibiting a difference of only 17 amino acids from 1137insC (CX46S380fs). CX46fs380 did not traffic properly and was retained within the Golgi compartment [18], resulting in a loss of function in the formation of gap and hemigap junctional channels [19]. Therefore, it is necessary to predict the function of CX46A397fs in the pathogenesis of congenital cataract.

## Methods

### Patient identification

A four-generation family with autosomal dominant cataract was examined at the First Affiliated Hospital of Zhengzhou University, Henan, China. Both affected and unaffected individuals were subject to detailed ophthalmic examinations, including A-scan and B-scan ultrasonography; ocular motility assessment; and visual acuity, intraocular pressure, papillary, fundus, and slit-lamp examinations. The relevant phenotypes were documented using slit-lamp photography, and no other history of disease was examined. Eight members of the ADCC family participated in the study, and seven were diagnosed as affected. EDTA-anticoagulated venous blood samples were collected from all participants. Genomic DNA was extracted from peripheral blood lymphocytes through standard procedures using FlexiGene DNA kits (Qiagen Inc., Hilden, Germany). This study was approved by the appropriate ethical review committees and conducted in accordance with the Declaration of Helsinki principles.

### Exome sequencing

**Figure 1:**
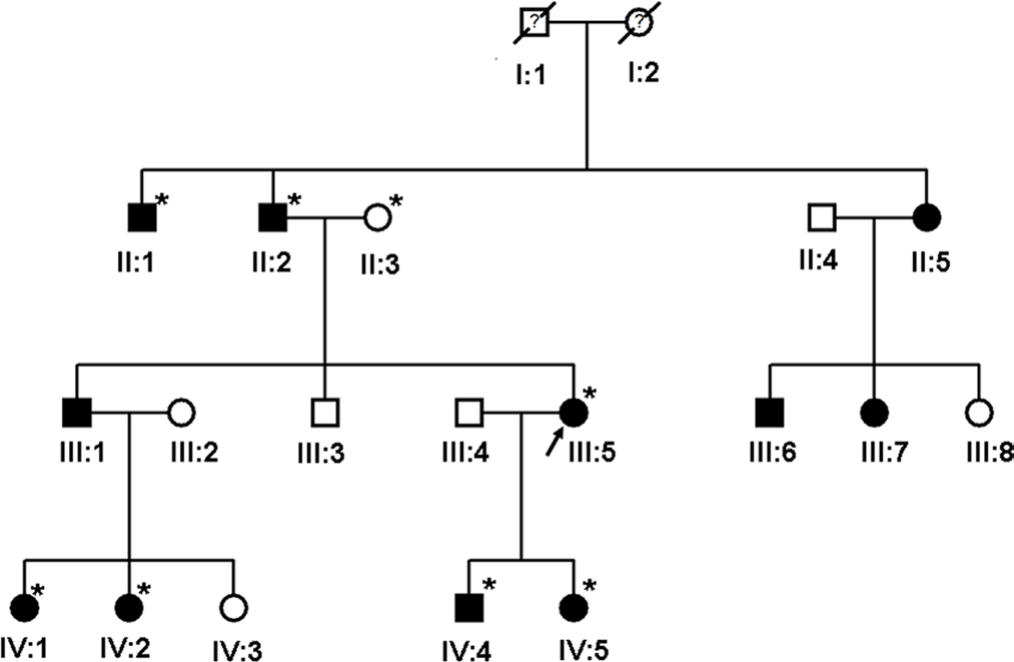
Pedigree of family with autosomal dominant coralliform cataract. The photographs of the proband (III:5) and IV:1 were taken after birth. They were diagnosed with coralliform cataract shortly after birth. This type of cataract turbidity changes little with age and was congenital and nonprogressive. These patients denied eye trauma operation history. No other abnormalities were found except for the coralliform cataract after external ocular examination. Symbols marked with asterisks represent individuals who were analyzed.

The identity of the mutant gene for congenital cataract was not initially known, so two unoperated affected individuals (III-5, IV-1) and one unaffected individual (II-3) in the ADCC family (Figure 1) were selected for exome sequencing. The methods of exome sequencing were outlined in previous literature [20,21]. Sequencing was performed with 100bp paired-end reads on a HiSeq2000 (Illumina, San Diego, CA). Reads were aligned to the human reference genome with BWA. Variants were called with GATK and annotated with Annovar. Pathogenicity predictions for variants were obtained from Annovar and the scale-invariant feature transform (SIFT) algorithm. Sequencing coverage depth was calculated using BEDTools and genomic coordinates provided by Illumina. Exome capture was carried out using TruSeq Exome Enrichment kit (Illumina) according to the manufacturer's protocols. Briefly, genomic DNA samples were randomly fragmented by Covaris with a base-pair peak of 200-300 bp for the resulting fragments, and adapters were then ligated to both ends of the fragments. The adaptor-ligated templates were purified using Agencourt AMPure XP beads, and fragments with insert size 300-400 bp were excised. Extracted DNA was amplified by ligation-mediated PCR, purified and hybridized to the SureSelect Biotinylated RNA Library (BAITS) for enrichment. Hybridized fragments bound to the strepavidin beads, whereas nonhybridized fragments were washed out after 24 hr. Captured ligation-mediated PCR products were subjected to an Agilent 2100 Bioanalyzer to estimate the magnitude of enrichment. Each captured library was then loaded on a HiSeq 2000 platform, and paired-end sequencing was performed with read lengths of 100 bp, which provided at least 50× average coverage depth for each sample. Raw image files were processed by Illumina base-calling Software 1.8 for base calling with default parameters.

### Mutation identification by Sanger sequencing

Per the results of exome sequencing, we used polymerase chain reaction (PCR) to amplify the mutation area of *GJA3* from genomic DNA (the forward primer 5’-AGC CCT CAG CGA CCA GAT T-3’ and the reverse primer 5’-CAG TCC GCC AAG CTC TAC AA-3’, annealing temperature: 57 °C), and the reverse primer was used as sequencing primer. The sequencing was performed using an ABI BigDye Terminator Cycle Sequencing Kit (Applied Biosystems, CA) on an ABI PRISM 3130 DNA Analyzer (Applied Biosystems), and data were analyzed using sequence analysis software, version 3.4.1 (Applied Biosystems). Genomic DNA from all affected and unaffected members of the ADCC family, and from 100 unrelated normal controls, were analyzed. Sequence data were compared with the GJA3 reference sequence (GenBank NM_174917.2) using Sequencher 4.10.1 (Gene Codes Corp.). Nucleotide numbering reflects complementary DNA (cDNA) numbering, with +1 cor-responding to the A of the ATG translation initiation codon in the reference sequence).

### Bioinformatics analysis

DNAMAN software was used to predict the functional effect of the identified (*GJA3* fs397) mutation on the conservation of GJA3 protein in different species. The effects of CX46S380fs and CX46A397fs mutation on the secondary structure of GJA3 were predicted using the Garnier-Osguthorpe-Robson (GOR) method, and the alignment of these two frameshift mutation sequences was predicted using the webpage Embnet.

## Results

### Clinical findings

**Figure 2:**
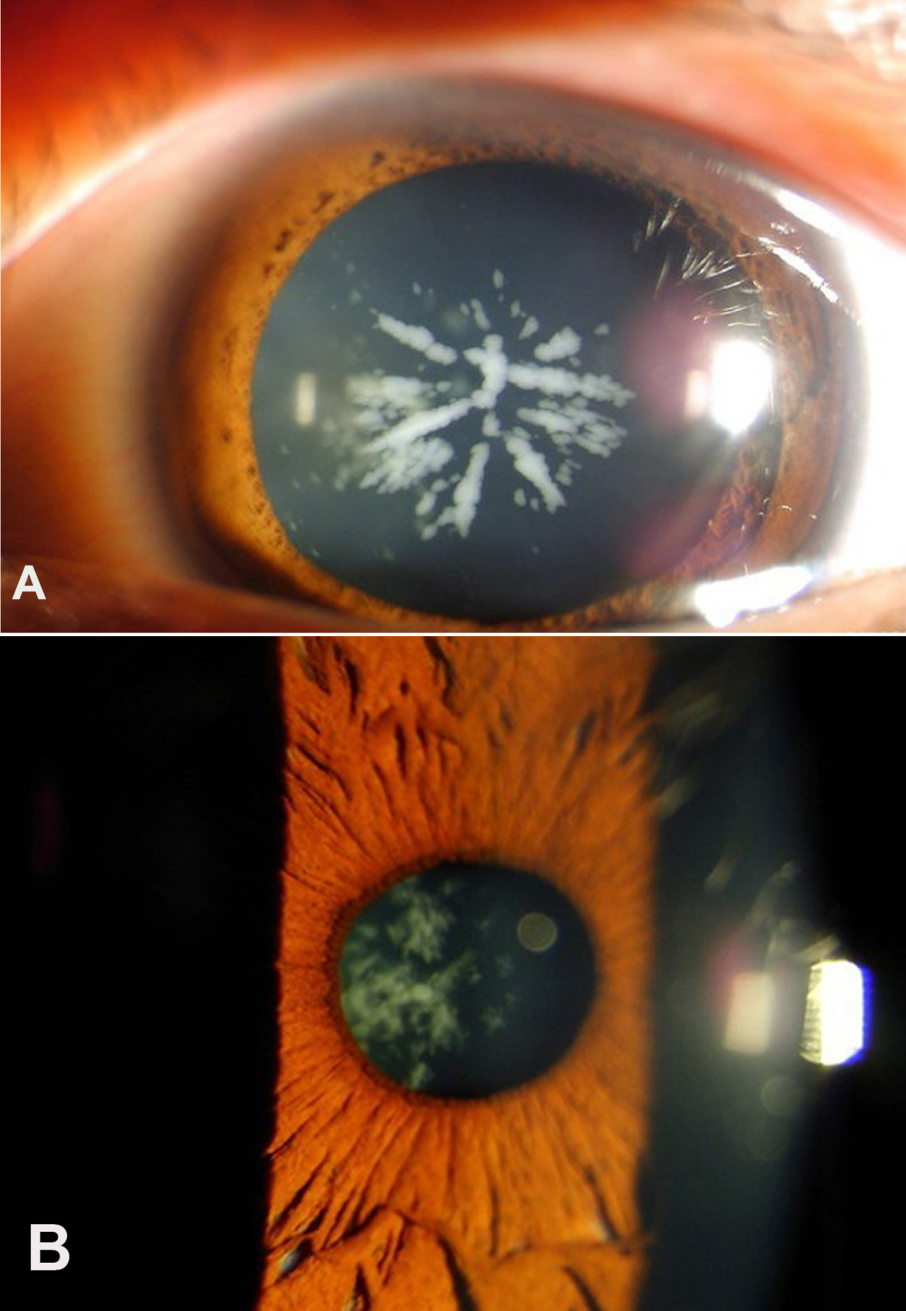
Slit-lamp photograph of two affected individuals with approximately 20/40 visual acuity in this four-generation Chinese family. **A**: The left eye of III:5 demonstrated coralliform cataract characterized with central radial lenticular opacities with a resemblance to sea coral. **B**: The right eye of IV:1 exhibited snowball-shaped clumps of crystals (coralliform or crystalline cataracts).

After reviewing the clinical examinations and hospital records of a four-generation family diagnosed with congenital cataract (Figure 2), seven affected individuals were diagnosed with congenital coralliform cataracts, exhibiting an almost sea coral or crystalline cataract appearance. The left eye of III:5 demonstrated coralliform cataract characterized with central radial lenticular opacities resembling sea coral (Figure 2A). The right eye of IV:1 showed snowball-shaped clumps of crystals (coralliform or crystalline cataracts; Figure 2B).

### Gap junction alpha 3 (*GJA3*) mutation analysis

**Figure 3:**
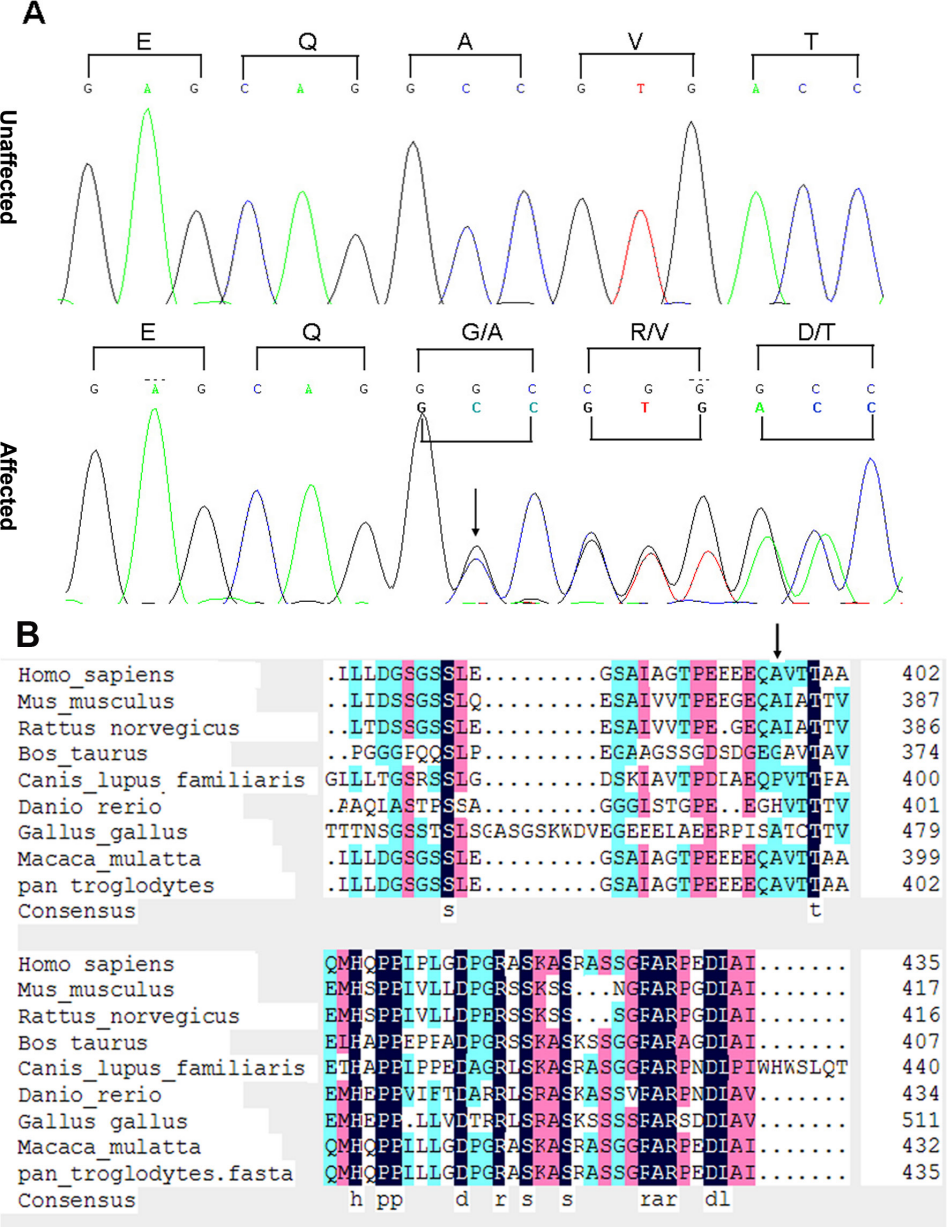
DNA sequence chromatograms and cosegregation analysis showing the cytosine insertion at position 1361 of the cDNA (p.1361insC) of gap junction alpha 3 (*GJA3*). **A**: A portion of the *GJA3* DNA sequence in all affected and unaffected individuals of the autosomal dominant congenital cataract (ADCC) family. The sequence (antisense strand) demonstrates an insertional mutation in codon 397 in the affected individuals. Codons are marked by brackets and amino acids indicated above. **B**: Sequence alignment of the intercellular carboxy terminus of reference GJA3 from different species. The black bars highlight the conserved amino acids. Thirteen amino acids are showed to be highly conserved in the C-terminus of the GJA3 protein in different species. The mutation site of Ala 397 (indicated by an arrowhead) in human GJA3 protein only shares 67% homology with those of other species.

After all candidate genes were screened, a cytosine insertion after coding nucleotide 1361(c.1361_1362insC) was found in the *GJA3* gene. This novel insertion mutation causes a frameshift immediately after codon 397 (Figure 3A), and has been confirmed to cosegregate with the ADCC phenotype in all affected family members only. These mutations were not observed in any current database, including the HapMap and 1000 Genomes projects. Thus, the mutation is unlikely to represent single-nucleotide polymorphism.

**Figure 4:**
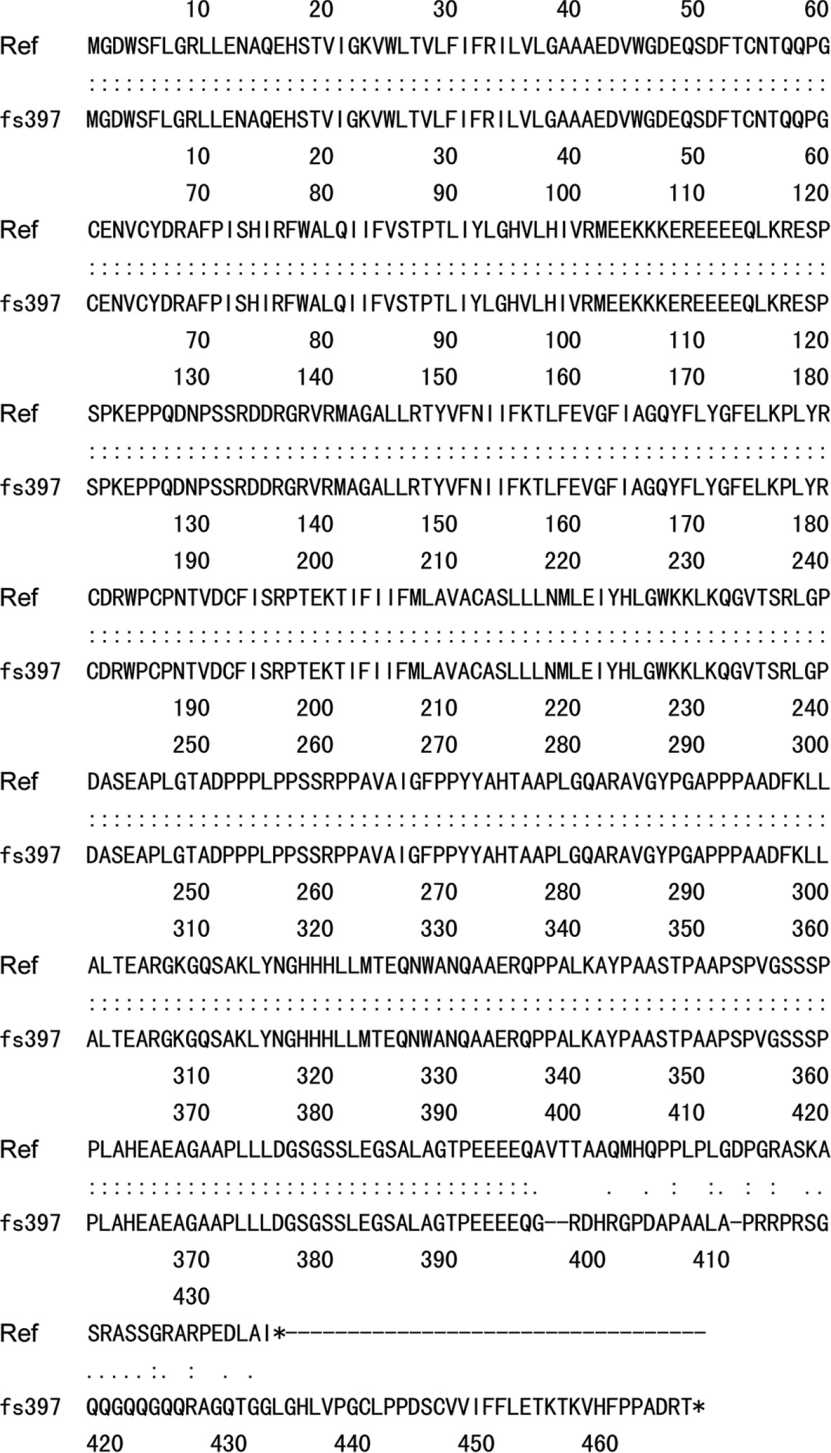
The alignment of the deduced *GJA3* amino acid sequence of reference and CX46A397fs mutant. The 1361 insC mutation causes a frameshift at amino acid 397 in the cytoplasmic C-terminus. In-frame translation stop codons are indicated by asterisks. Ref: reference.

The 1361 insC (Ala397Glyfs**×**71) mutation induces a significant frameshift at amino acid 397 in the *GJA3* C-terminus, resulting in the mistranslation of the final 40 amino acids of the reference protein and the addition of 31 amino acids to the C-terminus of the mutant protein before an in-frame translation stop codon is detected (Figure 4). Alignment of the 71 novel amino acids in the SWISS-PROT database failed to detect significant homology with other known proteins, so the homology model of the mutant GJA3 protein is difficult to generate.

### Bioinformatics analysis results

**Figure 5:**
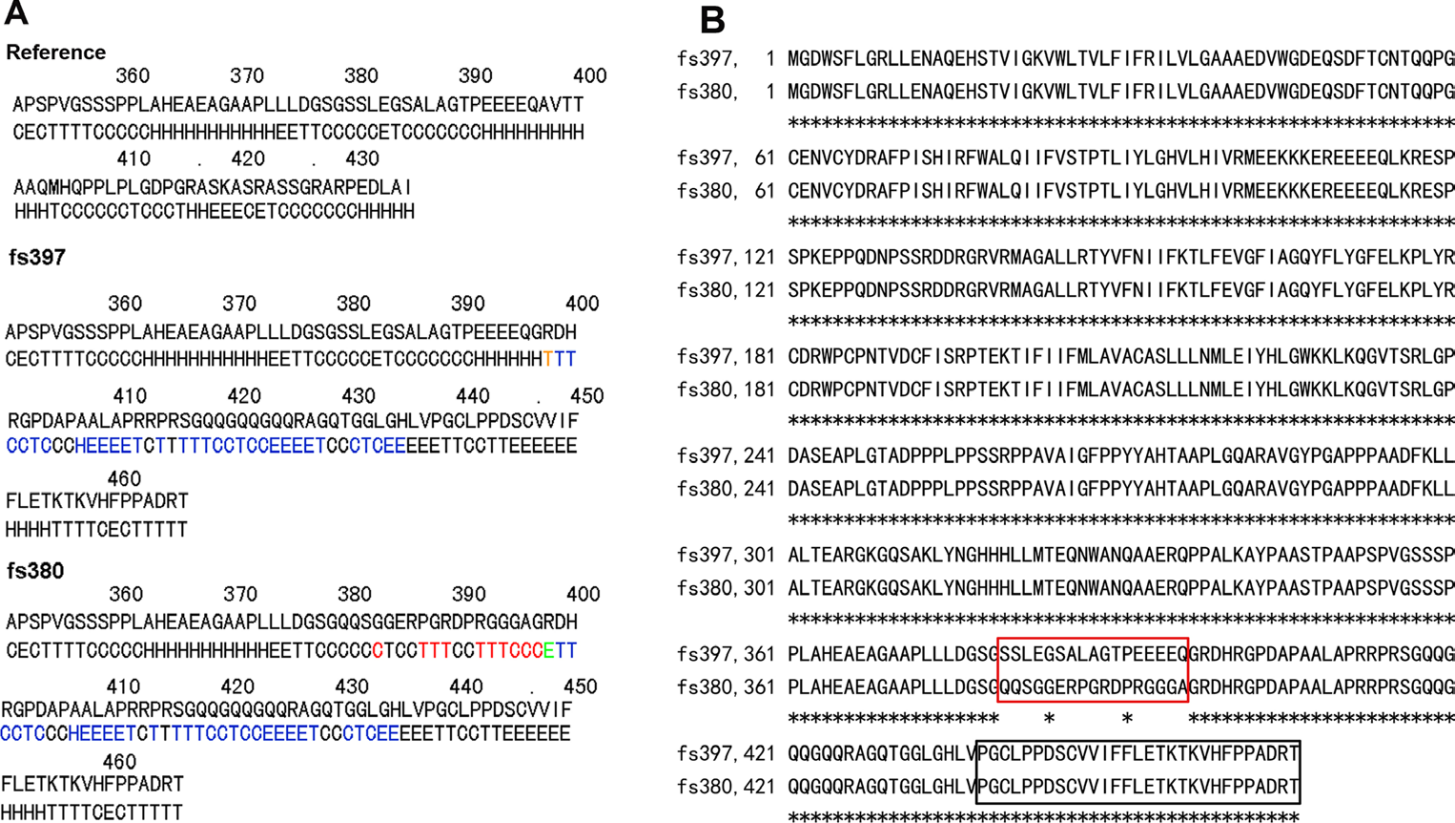
The effect of CX46A397fs mutation on the secondary structure of GJA3 protein. **A**: The frameshift prediction results using the GOR method. The changes in secondary structure specific in CX46S380fs are indicated in red (along with a specific change in the secondary structure of one out of the 17 amino acids); the changes in secondary structure shared by CX46A397fs and CX46S380fs are indicated in blue. One amino acid at the 398^th^ site of CX46A397fs and CX46S380fs mutant possesses a secondary structure different from that of the reference, indicated in brown and blue, respectively. **B**: The alignment of CX46A397fs and CX46S380fs mutant protein sequences demonstrated 96.8% identity in 466 amino acid residues. The 17 amino acids that differ between CX46S380fs and CX46A397fs are blanketed in red. The 29 amino acids that are shown to impair the function of CX46380fs mutant are blanketed in black.

Thirteen amino acids (marked by black bars) after residue 397 of human GJA3 are predicated to be 100% homologous with those of other species, suggesting the high conservation of these amino acids in different species (Figure 3B). The CX46A397fs mutation results in an obvious mistranslation of the final 40 (including the 13 highly conserved) amino acids in the GJA3, and would thus be expected to significantly influence protein function and induce disruption of the protein’s cytoplasmic C-terminus. Using the GOR method, the predicted results for secondary structure suggest that the mutant CX46A397fs differs from CX46S380fs by only 17 amino acids. Within the 17 mutated amino acids, prediction results indicate that there is one “C” (representing random coil) replaced by “E” (beta sheet) at amino acid 383, 3 “T’s” (beta turns) replaced by 3 “C’s” at amino acids 387–389, and 3 “T’s” along with 2 “C’s” replaced by 5 “H’s” (alpha helices) at amino acids 392–396. There are also 7 “C’s” and one “T” that remain unchanged. However, there is a “C” to “H” variation at amino acid 397 and an “E” to “T” variation at amino acid 398; these are not included in the 17 amino acid count (Figure 5A). The CX46A397fs and CX46S380fs are parallel starting from the 397 glycine, with the 17 amino acid mutation from 380 to 396 forming the only difference between these two mutants. The alignment of the CX46A397fs and CX46S380fs mutant protein sequences shows 96.8% identity in 466 amino acid residues (Figure 5B).

## Discussion

In this study, we localized the candidate gene of a four-generation Chinese congenital coralliform cataract pedigree in the *GJA3* locus and identified a novel insertional mutation (c.1361_1362insC) of *GJA3*. This insertion mutation is the second reported mutation of *GJA3* to have been localized to the intracellular C-terminus, indicating that the C-terminus plays an essential role in *GJA3* gene function.

Up to now,at least 15 mutations of *GJA3* have been reported to cause ADCC [6-16]. Most of these reported mutations have been mapped in the NH2-terminal end, the first transmembrane domain (M1), the first extracellular domain (EC-1),the first extracellular loop (EL1) or the second extracellular loop (EL2; Table 1) and predicated to damage the GJA3 function.

**Table 1:** The summary of previous studies of congenital cataract associated with GJA3

**Mutation**	**Amino Acid Changes**	**Location**	**Cataract Type**	**Family Origin**	**Reference**
c.7G>T	p.D3Y	NH2-terminus	Zonular pulverulent	Hispanic Central American	[9]
c.32T>C	p.L11S	NH2-terminus	Ant-egg	Danish	[11]
c.82G>A	p.V28M	First transmembrane domain (TM1)	Variable	Indian	[12]
c.96C>A	p.F32L	First transmembrane domain (TM1)	Nuclear pulverulent	Chinese	[7]
c.98G>T	p.R33L	First transmembrane domain (TM1)	Embryonal nuclear granular	Indian	[10]
c.130G>A	p.V44M	First extracellular loop (EL1)	Bilateral nuclear	Chinese	[13]
c.134G>C	p.W45S	First extracellular loop (EL1)	Bilateral nuclear	Chinese	[14]
c.176C>T	p.P59L	First extracellular loop (EL1)	Nuclear punctate	American	[6]
c.188A>G	p.N63S	First extracellular loop (EL1)	Zonular pulverulent	Caucasian	[8]
c.226C>G	p.R76G	First extracellular loop (EL1)	Total	Indian	[12]
c.227G>A	p.R76H	First extracellular loop (EL1)	Pulverulent	Australian	[12,22]
c.260C>T	p.T87M	Second transmembrane domain (TM2)	Pearl box	Indian	[12]
c.427G>A	p.G143R	Topological domain	Coppock-like cataract	Chinese	[16]
c.560C>T	p.P187L	Second extracellular loop (EL2)	Zonular pulverulent	Caucasian	[17]
c.563A>C	p.N188T	Second extracellular loop (EL2)	Nuclear pulverulent	Chinese	[23]
c.563A>T	p.N188I	Second extracellular loop (EL2)	Zonular pulverulent	Chinese	[15]
c.1137insC	p.S380fs	COOH-terminus	Zonular pulverulent	Caucasian	[8]
c.1361insC	p.A397fs	COOH-terminus	coralliform	Chinese	This study

It is likely that reduced intercellular communication due to human *GJA3* mutations trigger the formation of cataracts, which may be explained by decreased translation and/or enhanced degradation of connexin 46 [19]. Due to the high similarity of CX46A397fs to CX46S380fs in protein sequence and secondary structure, we hypothesize that CX46A397fs may also play a similar function in the pathogenesis of congenital cataract.

CX46S380fs did not traffic properly and was retained within the Golgi compartment. The last 29 of the 87 novel amino acids generated by CX46S380fs were required for impaired trafficking and loss of function [22]. These 29 amino acids also appeared in the carboxy terminus of CX46A397fs (blanketed in black in Figure 5B). Taken together, the last 29 of the novel 71 amino acids generated by CX46A397fs are necessary for the pathogenesis of congenital cataract.

In this study, the 1361 insC (p. Ala397Glyfs×71) mutation results in a 210-nucleotide addition and a late-mature translation stop codon. As indicated in the Phospho Site Plus database, no modification sites are localized in the 397 amino acid site, so this mutation is not likely to influence posttranslational modifications, including phosphorylation of the GJA3 protein. However, 13 amino acids after the 397 residue of human GJA3 are predicated to be conserved in different species. This insertional mutation results in the disruption of the 13 conserved amino acids in the intracellular GJA3 carboxy terminus, which may significantly alter GJA3 function.

In conclusion, a novel insertional mutation of the *GJA3* gene was identified in a four-generation Chinese family with autosomal dominant congenital coralliform cataract. The abnormal carboxy terminus generated due to a frameshift in CX46A397fs is predicted to cause loss of function, which may be pivotal to the formation of congenital cataract.
